# Frailty and Emergency Surgery: Results of a Systematic Review and Meta-Analysis

**DOI:** 10.3389/fmed.2022.811524

**Published:** 2022-03-31

**Authors:** Tamas Leiner, David Nemeth, Peter Hegyi, Klementina Ocskay, Marcell Virag, Szabolcs Kiss, Mate Rottler, Matyas Vajda, Alex Varadi, Zsolt Molnar

**Affiliations:** ^1^Institute for Translational Medicine, Medical School, University of Pecs, Pecs, Hungary; ^2^Anaesthetic Department, Hinchingbrooke Hospital, North West Anglia NHS Foundation Trust, Huntingdon, United Kingdom; ^3^Centre for Translational Medicine, Semmelweis University, Budapest, Hungary; ^4^Division for Pancreatic Disorders, Heart and Vascular Center, Semmelweis University, Budapest, Hungary; ^5^Doctoral School of Clinical Medicine, University of Szeged, Szeged, Hungary; ^6^Department of Anesthesiology and Intensive Therapy, Szent Gyorgy University Teaching Hospital of Fejer County, Szekesfehervar, Hungary; ^7^Department of Anesthesiology and Intensive Therapy, Poznan University of Medical Sciences, Poznan, Poland; ^8^Department of Anesthesiology and Intensive Therapy, Semmelweis University, Budapest, Hungary

**Keywords:** frail adults, emergency surgery, mortality, Clinical Frailty Scale, meta-analysis

## Abstract

**Background:**

Frailty, a “syndrome of loss of reserves,” is a decade old concept. Initially it was used mainly in geriatrics but lately its use has been extended into other specialties including surgery. Our main objective was to examine the association between frailty and mortality, between frailty and length of hospital stay (LOS) and frailty and readmission within 30 days in the emergency surgical population.

**Methods:**

Studies reporting on frailty in the emergency surgical population were eligible. MEDLINE (via PubMed), EMBASE, Scopus, CENTRAL, and Web of Science were searched with terms related to acute surgery and frail^*^. We searched for eligible articles without any restrictions on the 2nd of November 2020. Odds ratios (OR) and weighted mean differences (WMD) were calculated with 95% confidence intervals (CI), using a random effect model. Risk of bias assessment was performed according to the recommendations of the Cochrane Collaboration. As the finally selected studies were either prospective or retrospective cohorts, the “Quality In Prognosis Studies” (QUIPS) tool was used.

**Results:**

At the end of the selection process 21 eligible studies with total 562.070 participants from 8 countries were included in the qualitative and the quantitative synthesis. Patients living with frailty have higher chance of dying within 30 days after an emergency surgical admission (OR: 1.99; CI: 1.76–2.21; *p* < 0.001). We found a tendency of increased LOS with frailty in acute surgical patients (WMD: 4.75 days; CI: 1.79–7.71; *p* = 0.002). Patients living with frailty have increased chance of 30-day readmission after discharge (OR: 1.36; CI: 1.06–1.75; *p* = 0.015).

**Conclusions:**

Although there is good evidence that living with frailty increases the chance of unfavorable outcomes, further research needs to be done to assess the benefits and costs of frailty screening for emergency surgical patients.

**Systematic Review Registration:**

The review protocol was registered on the PROSPERO International Prospective Register of Systematic Reviews (CRD42021224689).

## Introduction

Emergency surgery carries higher risk of mortality and morbidity. Appropriate risk assessment, attentive decision-making and carefully selected interventions are the cornerstones of a patient centered management (1, 2). To achieve the best possible outcome multiple factors need to be considered. It is well-known that ASA (PS) (American Society of Anesthesiologists Physical Status Classification System) score 3 or above is an independent predictor of unfavorable outcomes not just in elective but more so in emergency surgery (3). Furthermore, older age, male sex, peritoneal contamination, use of oral anticoagulant, need for blood transfusion, hypoalbuminemia and electrolyte abnormalities (potassium and sodium) have a negative influence on the success rate of emergency surgical interventions (4, 5).

Frailty, a “syndrome of loss of reserves,” is more than decade old concept (6). Initially it was used mainly in geriatrics but lately its use has been extended into other specialties including surgery. A meta-analysis demonstrated that frail surgical patients had a higher risk of readmission and increased risk of mortality (7). In a very recent preliminary analysis McIntyre et al. found that frailty was associated with worse surgical outcomes following chronic subdural hemorrhage, but the clinical utility of the frailty scores remained unclear (8). In a large prospective multinational study Haas et al. demonstrated that frailty was significantly associated with an increased 6-month mortality in elderly intensive care patients admitted with sepsis (9).

Several large retrospective and small prospective cohort studies have been published reporting on the association between frailty, mortality and post-operative complications (10–12). In a relatively large, prospective multicenter observational trial from the United Kingdom Parmar et al. reported that frailty was present in 20% of older adults undergoing emergency laparotomy and was independent of age (10).

The aim of our systematic review and meta-analysis was to investigate whether frailty, indicated by any validated score in the emergency surgical patient population is associated with increased chance of mortality (in hospital, 30-day, 90-day, and 12 month), 30-day readmission and prolonged hospital stay.

## Methods

### Protocol, Registration, and Reporting

This systematic review and meta-analysis is reported in accordance with the Preferred Reporting Items for Systematic Reviews and Meta-Analyses Statement (PRISMA) (13). The review protocol was registered on the PROSPERO International Prospective Register of Systematic Reviews and adhered to it completely (CRD42021224689).

### Search Strategy

MEDLINE (via PubMed), EMBASE, Cochrane Central Register of Controlled Trials (CENTRAL), Scopus, and Web of Science were searched for eligible articles without any restrictions on the 2nd of November 2020. We also scanned the reference lists of included studies or relevant reviews identified through the search for further articles. Re-run searches prior final analysis have not been carried out. The following search term was used: (frailty OR frail) AND (emergent OR urgent) AND (surgery OR surgical OR operation).

### Selection and Eligibility Criteria

We formulated our clinical question using the PICO format (Supplementary Materials) and selected clinical studies reporting on frailty within the emergency surgical patient group (aged over 18 years old and admitted to hospital with a general surgical complaint, including those undergoing surgery and those managed conservatively). Any validated score or method cited in the literature or explained in detail in the article's method section were accepted. Studies were included in the systematic review if at least one of the following outcomes could be extracted: 30-day mortality, defined as death during the 30-day period following emergency general surgical admission or primary intervention; 90-day mortality, defined as death during the 90-day period following emergency general surgical admission or primary intervention, in-hospital mortality, length of hospital stay and 30-day hospital readmission. Randomized controlled trials, prospective or retrospective cohorts, and case-control studies, independently of the number of included patients, were included. Unpublished preprints, letters, editorials, review articles, case reports, and case series (≤ 10 patients) were excluded.

After the removal of duplicates using a reference management software (EndNote X9, Clarivate Analytics), TL and MV independently screened titles, abstracts, and then full texts against predefined eligibility criteria. Inter-rater reliability was determined by Cohen's kappa coefficient, where values 0.61–0.80 indicate substantial and 0.81–1.00 indicate almost perfect or perfect agreement (14). Discrepancies were resolved by a third review author (KO). From those studies that had either proven or suspected overlapping population we either included the one with the largest sample size or the most recent. If a study used more than one score to assess frailty, we opted to use the one which was used by other researchers too. Outcomes reported by at least two studies using the same frailty score comparing identical frailty subgroups were included in the meta-analysis. All other eligible studies and data were incorporated into the qualitative synthesis.

### Data Extraction

Two review authors (MV) and (TL) independently extracted data into a standardized data collection form (Microsoft Excel). The following data were extracted from each eligible article: first author, publication year, country of study, study design, number of patients in each comparison group, their baseline characteristics (sex, age), type of frailty score used, and available outcome parameters (in-hospital mortality, 30-day mortality, 90-day mortality, length of hospital stay and 30-day hospital readmission). Data extraction was validated by a third review author (KO).

### Risk of Bias Assessment

Risk of bias assessment was performed according to the recommendations of the Cochrane Collaboration. Two independent investigators (LT and MV) assessed the quality of the studies included. Any disagreement was resolved based on consensus. As the studies finally selected were either prospective or retrospective cohorts, the “Quality In Prognosis Studies” (QUIPS) tool was used (15).

### Statistical Analysis

Methods recommended by the working group of the Cochrane Collaboration were used for data synthesis (16). We collected data on frail and non-frail patients from the selected studies and sorted them either to “event” or “non-event” groups. The two groups were compared and odds ratios with 95% confidence interval were calculated. We used crude odds ratio if no raw data was presented in the original article. Due to the difference in precision of included studies a random effect model was used, and the results were displayed on Forest Plots. Weighted mean differences (WMD) of length of hospital stay (LOS) were calculated, and the values between the frail and non-frail groups were compared.

Heterogeneity was tested by the Cochran's Q test and Higgins' *I*^2^ indicator (17, 18). The Q statistics were computed as the weighted sum of individual study effects' squared deviations from the pooled effect, with the weights being used in the pooling method; *p*-values were obtained by comparing the test statistics with a chi-square with k-1 degrees of freedom (where k was the number of studies). A *p*-value of < 0.10 was considered suggestive of significant heterogeneity. The *I*^2^ index corresponds to the percentage of the total variability across studies that is due to heterogeneity. Based on Cochrane's handbook, a rough classification of its value is the following: might not be important (0–40%), may represent moderate heterogeneity (30–60%), may represent substantial heterogeneity (50–90%) and considerable heterogeneity (75–100%) (19).

Where it was possible, we performed subgroup analysis as well, according to the measurements of frailty such as Clinical Frailty Scale (CFS), Emergency General Surgery Frailty Index (EGSFI) and Triage Risk Stratification Tool (TRST) and G8 [Geriatric screening tool (G8)].

All the statistical analyses were performed using Stata IC (version 16, StataCorp LLC, 4905 Lakeway Drive College Station, Texas 77845-4512, USA).

## Results

**Figure 1:**
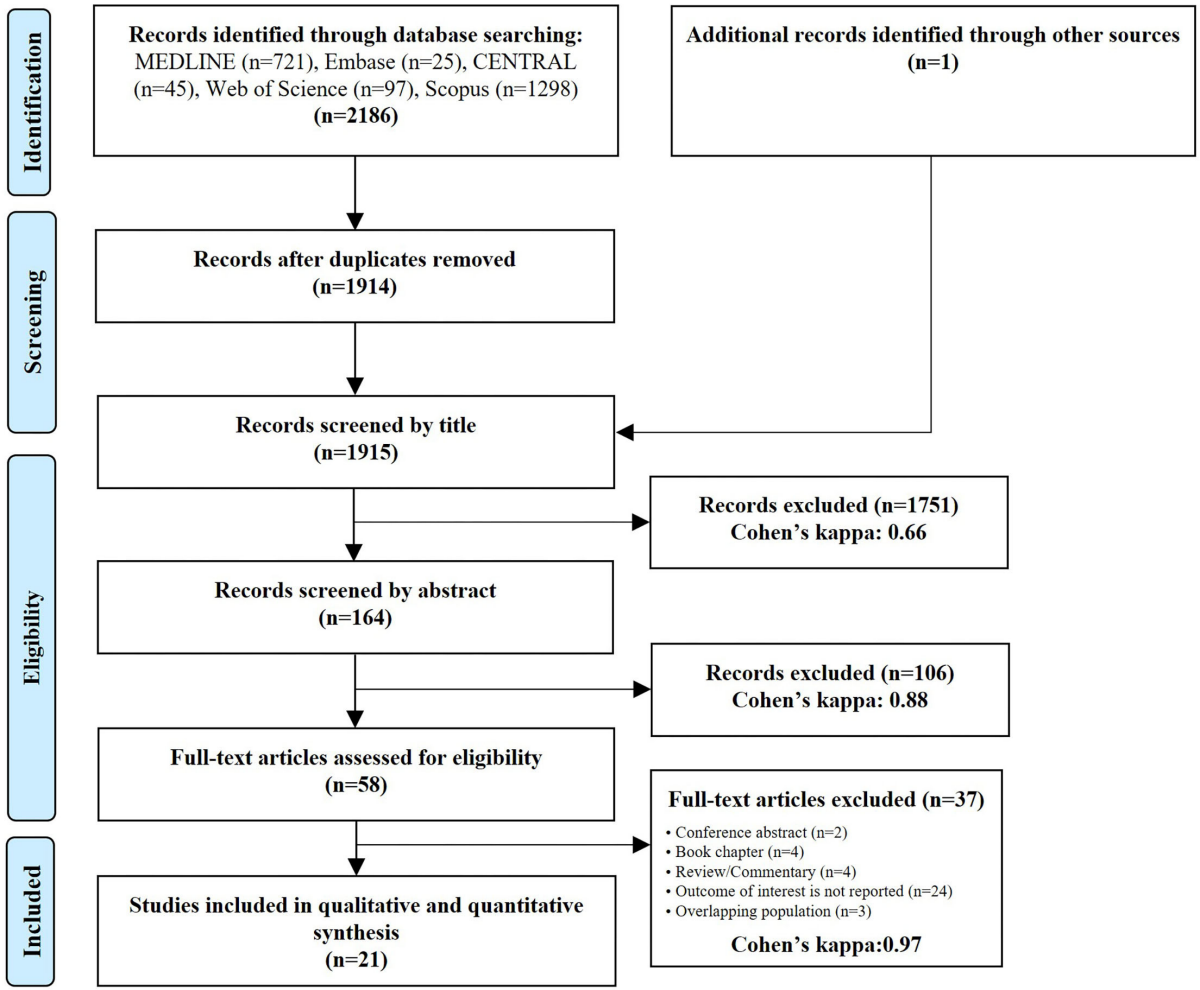
PRISMA flowchart of study selection.

Our systematic search yielded 2,186 records and one more study was identified through reference lists. After removal of duplicate records 1915 records remained and were screened by title, 164 by abstract and 58 by full text. At the end of the selection process 21 eligible studies from 8 countries were included into the review. The results of our search and selection are detailed in the PRISMA Flow Diagram shown in Figure 1. Characteristics of the included studies are summarized in Table 1.

**Table 1:** Characteristics of included studies.

**Publication**			**Setting**						
**First author and year of publication**	**Design**		**Recruitment/** **examined period**	**Country**	**No. of centers**	**No. of patients**	**Frailty definition /score used**	**Inclusion criteria**	**Exclusion criteria**
	**Prosp./** **Retrosp**.	**Database**							
Arteaga (20)	P	No	09/2017–04/2019	Spain	1	92	CFS-9 FRAIL score TRST SHARE-FI	Patients older than 70 years, abdominal emergency surgery.	Under 70 years, patients with moderate to severe cognitive deterioration and patients with terminal illness, defined as a life expectancy of <6 months.
Goeteyn (21)	P	No	07–11/2016	Belgium	1	98	CFS-7	Patients older than 65 admitted to a general surgery ward from the emergency department were eligible for inclusion.	Not reported
Hewitt (22)	P	No	05–06/2013	UK	3	325	CFS-7	Patients aged over 65 years of age admitted to the acute general surgical admission units.	Not reported
Hewitt (23)	P	No	07–10/2014	UK	5	411	CFS-7	Patients aged 65 years and emergency general surgical admissions.	Not reported
Hewitt (12)	P	No	05–07/2015 and 06–08/2016	UK	6	2,279	CFS-7	patients aged over 18 years old admitted with a general surgical complaint, including those undergoing surgery and those managed conservatively	excluded if they had an urological, gynecological or vascular diagnosis
Jokar (24)	P	No	2013–2014	USA	1	60	EGSFI	EGS patients 65 years or older with a surgical procedure and at least one day of hospital admission	patients who refused to consent or in whom FI cannot be calculated secondary to an altered mental status and unavailability of family historians
Joseph (25)	P	No	10/2012–03/2014	USA	1	220	Rockwood FI 50	EGS patients with age ≥65 years who underwent a procedure in the operating room	Not reported
Kenig (26)	P	No	01/2013 and 07/2014	Poland	1	184	VES-13 TRST G8 GFI Rockwood FI Balducci	Patients 65 years of age or older, needing emergency abdominal surgery and treated surgically within 24 h after admission.	Patients that were unable to give informed consent, those that needed immediate operation, with incarcerated hernia with no need for laparotomy and operated > 24 h after admission were excluded.
Kenig (27)	P	No	06/2014 and 12/2015	Poland	1	60	GA	Patients over 65 years of age with inclusion criteria for the emergency patients were acute cholecystitis according to the 2013 Tokyo Guidelines symptomatic gallstone disease, acute cholecystitis requiring elective or emergency surgery.	Patients who were unable to give consent or answer the GA questions were excluded.
Kenig (28)	P	No	01/2013 and 12/2016	Poland	1	315	G8	Patients 65 years of age or older, needing emergency abdominal surgery within 24 h after admission.	Patients with no need for laparotomy (simple incarcerated inguinal/femoral hernia, patients with abdominal wall infections), acute pancreatitis, other emergency patients managed endoscopically, requiring only diagnostic laparoscopy or operated >24 h after admission were excluded.
Khan (29)	P	No	2014–2016	USA	1	326	EGSFI	all geriatric patients (age 65 y or older) who had an emergency surgical evaluation by the ACS service and had surgical intervention	elective general surgery patients, those transferred from other facilities, and those who died within 24 h after surgery
Lee (30)	R	Medicare	01/01/2008–31/12/2014	USA		468,459	CFI	Patients aged 65 years or older with at least 12 months of continuous Medicare enrolment before a qualifying EGS procedure were included.	Not reported
Li (31)	P	EASE study	01/2014 and 09/2015	Canada	2	322	CFS-9	Patients aged 65 years or older who survived emergency abdominal surgery.	Patients who required assistance with 3 or more activities of daily living, underwent palliative or trauma surgery, or were transferred from another ward or hospital were excluded.
McIsaac (32)	R	ICES	04/2002–03/2014	Canada	NA	77,184	ACG (J Hopkins U)	All residents of Ontario who were older than 65 years of age on the date of their first EGS procedure.	Patients residing in long-term care facilities before hospital admission were excluded.
Mahmooth (33)	P	No	05–09/2018	USA	1	272	EGSFI RAI-C Katz index	Participants were eligible if they were under the care of the ACCS service for at least 48 h, were not intubated or sedated, and were able and willing to provide information for the frailty assessments.	Patients with altered mental status were included if authorized family members or caretakers were available to provide information.
Parmar (10)	P	ELF Study	20/03–19/06/2017	UK	49	937	CFS-7	Older patients (defined as 65 y and older) undergoing emergency laparotomy	Not reported
Simon (11)	R	NSQIP	2012–2016	USA	NA	10,025	mFI-5	Patients aged at least 65 years who underwent emergency colorectal resection.	Elective, urgent or outpatient procedures, if the surgical approach was perineal, endoscopic, or unknown or outcomes of interest were missing
Vilches-Moraga (34)	P	No	09/2014–03/2017	UK	1	113	CFS-9	Patients aged 75 years or older undergoing emergency laparotomy.	Inpatient >90 days prior to the final date of data collection
Smart (35)	P	No	10/2014 and 03/2015	UK	1	169	CFS-7	Patients ≥40 years, emergency general surgical population	Not reported
Tan (36)	P	No	06/2016–02/2018	Singapore	1	109	mFFC mFI-11	Patients 65 years of age and above who underwent emergency abdominal surgery (including diagnostic laparoscopies and emergency abdominal wall hernia repairs).	Patients to remain an whose cognitive state precluded informed consent, and who had no next-of-kin to consent to the caregiver arm of the study, were excluded.
Zattoni (37)	P	No	12/2015 and 05/2016	Italy	1	110	fTRST	70 and older undergoing emergency abdominal surgery under general anesthesia.	Medical management only operated on for vascular, thoracic, gynecological, or urological conditions operations under locoregional anesthesia

*P, prospective; R, retrospective; ACG, Adjusted Clinical Group (Johns Hopkins); CFI, Claims-Based Frailty Index; CFS-7, 7-item Clinical Frailty Scale; CFS-9, 9-item Clinical Frailty Scale; EGSFI, Emergency General Surgery specific Frailty Index; fTRST, Flemish version of the Triage Risk Stratification Tool; FRAIL scale, 5-item FRAIL scale (Fatigue, Resistance, Ambulation, Illnesses, & Loss of Weight); G8, Geriatric screening tool (G8); GA, Geriatric Assessment; GFI, Groningen Frailty Index; mFFC, Modified Fried's Frailty Criteria; mFI-5, 5-item Modified Frailty Index; mFI-11, 11-item Modified Frailty Index; Rockwood FI, Rockwood Frailty Index; SHARE FI, Survey of Health, Aging and Retirement in Europe (SHARE) Frailty Instrument; VES-13, Vulnerable Elders Survey*.

### Risk of Bias

Risk of bias was assessed separately for mortality, 30-day readmission and LOS. Three studies had high over all risk of bias and other 3 had moderate risk of bias mainly due to confounding factors (Supplementary Figures S1–S3).

### 30-Day Mortality

**Figure 2:**
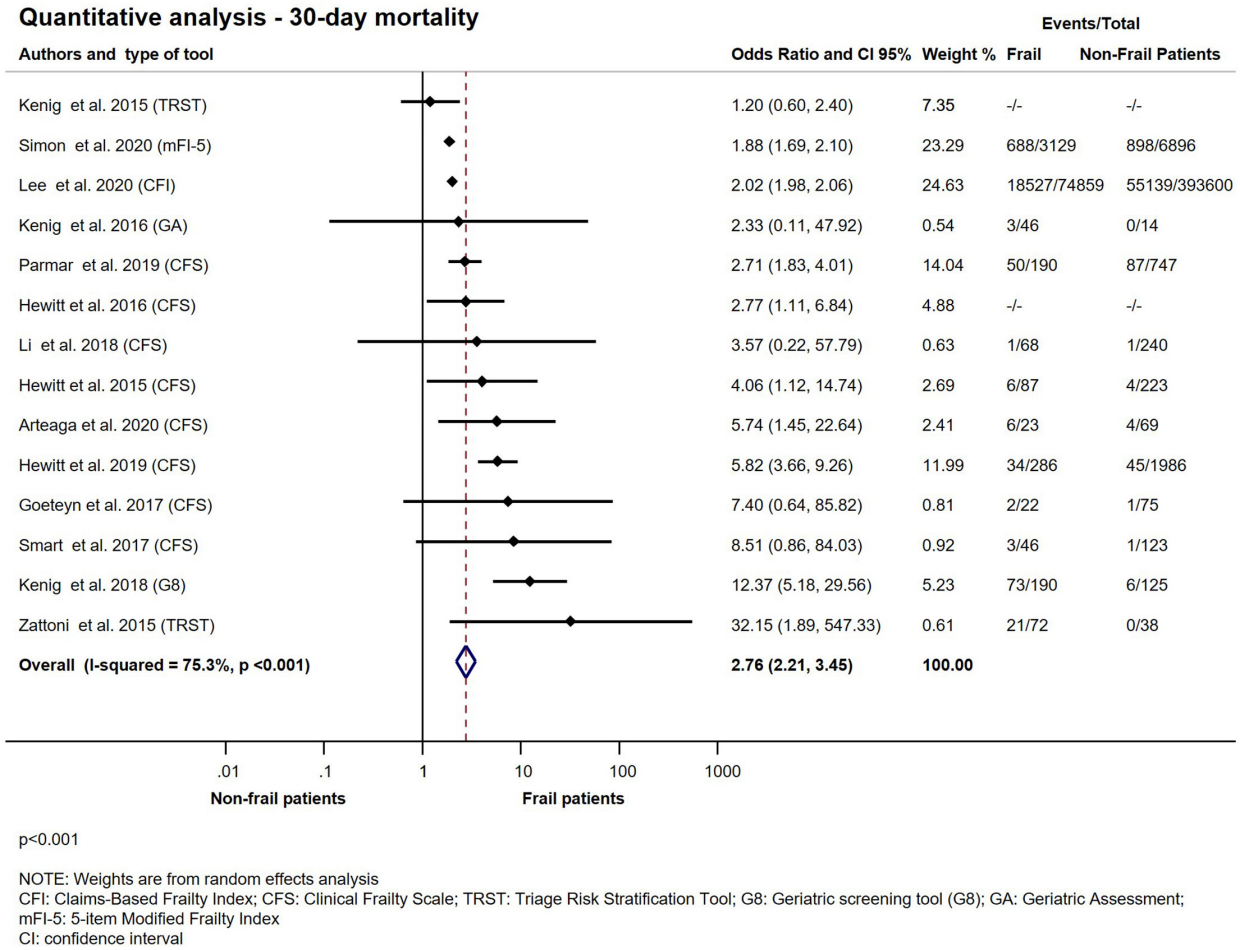
Quantitative analysis − 30-day mortality for emergency surgical patients living with frailty compared to non-frail patients. For patients living with frailty, the overall OR of 30-day mortality was 2.76 (2.21–3.45). From the study of Hewitt et al. (23) and Kenig et al. (26) crude OR was pooled with the ORs calculated from raw data. Note substantial heterogeneity.

**Figure 3:**
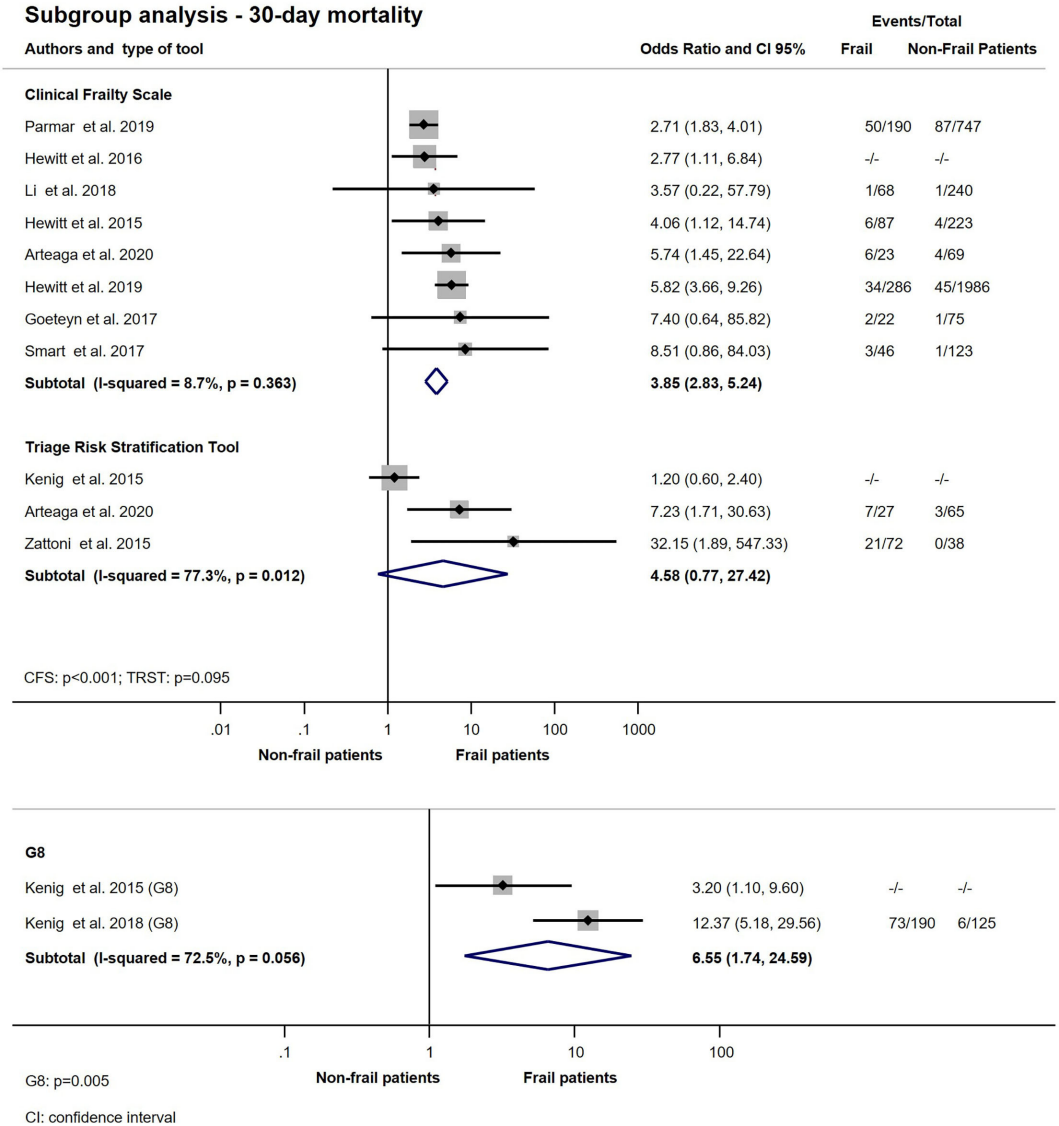
Subgroup analysis − 30-day mortality for emergency surgical patients living with frailty compared to non-frail patients. In the CFS subgroup frail patients (CFS > 4) have higher odds of 30-day mortality (OR: 3.85; CI: 2.83–5.24). In the frail TRST group (TRST > 2) the overall OR of 30-day mortality was 4.58; CI: 0.77–27.42. Frailty assessed with G8 showed that patients living with frailty had higher chance of dying within 30 days after hospital admission (OR: 6.55; CI: 1.74–24.59). Crude ORs were pooled with the ORs calculated from raw data. Note that heterogeneity might not be not important for CFS, but substantial for TRST and G8.

**Figure 4:**
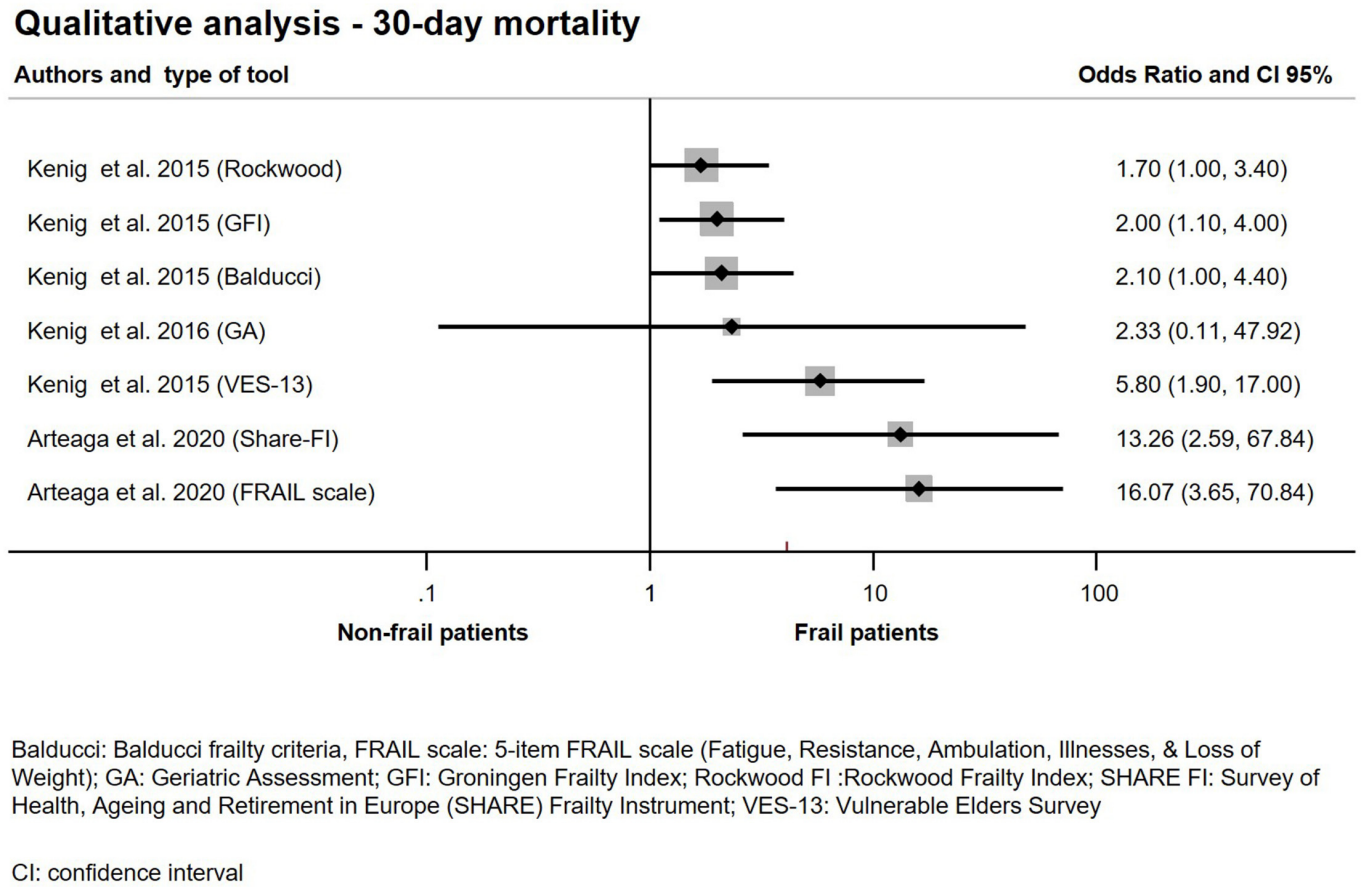
Qualitative analysis − 30-day mortality for emergency surgical patients living with frailty compared to non-frail patients.

A total of 14 studies with 483.722 participants, using 11 different frailty assessment methods examined the effect of frailty on 30-day mortality (10–12, 20–23, 26–28, 30, 31, 35, 37). Our analysis has shown that patients living with frailty have higher chance of dying within 30 days after an emergency surgical admission (OR: 2.76; CI: 2.21–3.45; *p* < 0.001) (Figure 2). Further quantitative synthesis was performed on studies using the 7- or 9-point version of the Clinical Frailty Scale (8 studies), the Triage Risk Stratification Tool (3 studies) and the Geriatric screening tool (G8) (10, 12, 20–23, 26, 28, 31, 35, 37). Frailty assessed with CFS indicated an increase in 30-day mortality approaching 4-fold values of that seen in non-frail population (OR: 3.85; CI: 2.83–5.24; *p* < 0.001), while TRST also showed similar results but did not reach statistical significance (OR: 4.58; CI: 0.77–27.42; *p* = 0.095). G8 was used for frailty assessment by the same authors in two small studies (26, 28). The subgroup analysis of these studies has shown a more than 6-fold increase chance of dying in the frail population (OR: 6.55; CI; 1.74–24.59; *p* = 0.005) (Figure 3). Two large retrospective cohort studies from the USA reported on a total of 478,484 patients (11, 30). Lee et al. has found significantly higher mortality in the frail group at 30 days: 24.0% in the mildly and 27.4% in the moderate to severely frail as compared to 11.1% in the non-frail group. Simon et al. used the five-item modified frailty index (mFI-5) score and demonstrated an increasing risk of 30-day mortality with frailty. Furthermore, in an adjusted regression model, after accounting for patient and procedure related factors, they found that frailty was still associated with increased risk of 30-day mortality. In two studies, Arteaga et al. and Kenig et al., the authors used multiple different frailty tools to assess frailty on the same emergency surgical population (20, 26). Living with frailty was associated with increased risk of 30-day mortality regardless which tool was used (Figure 4). In a small prospective study, also performed by Kenig et al. in 2016, patients who underwent emergency cholecystectomy were observed (27). A cumulative deficit model of frailty, Geriatric Assessment (GA) was used. Out of total 60 patients recruited to the study, 46 were living with frailty and all mortality was observed within this group.

### Hospital Mortality

**Figure 5:**
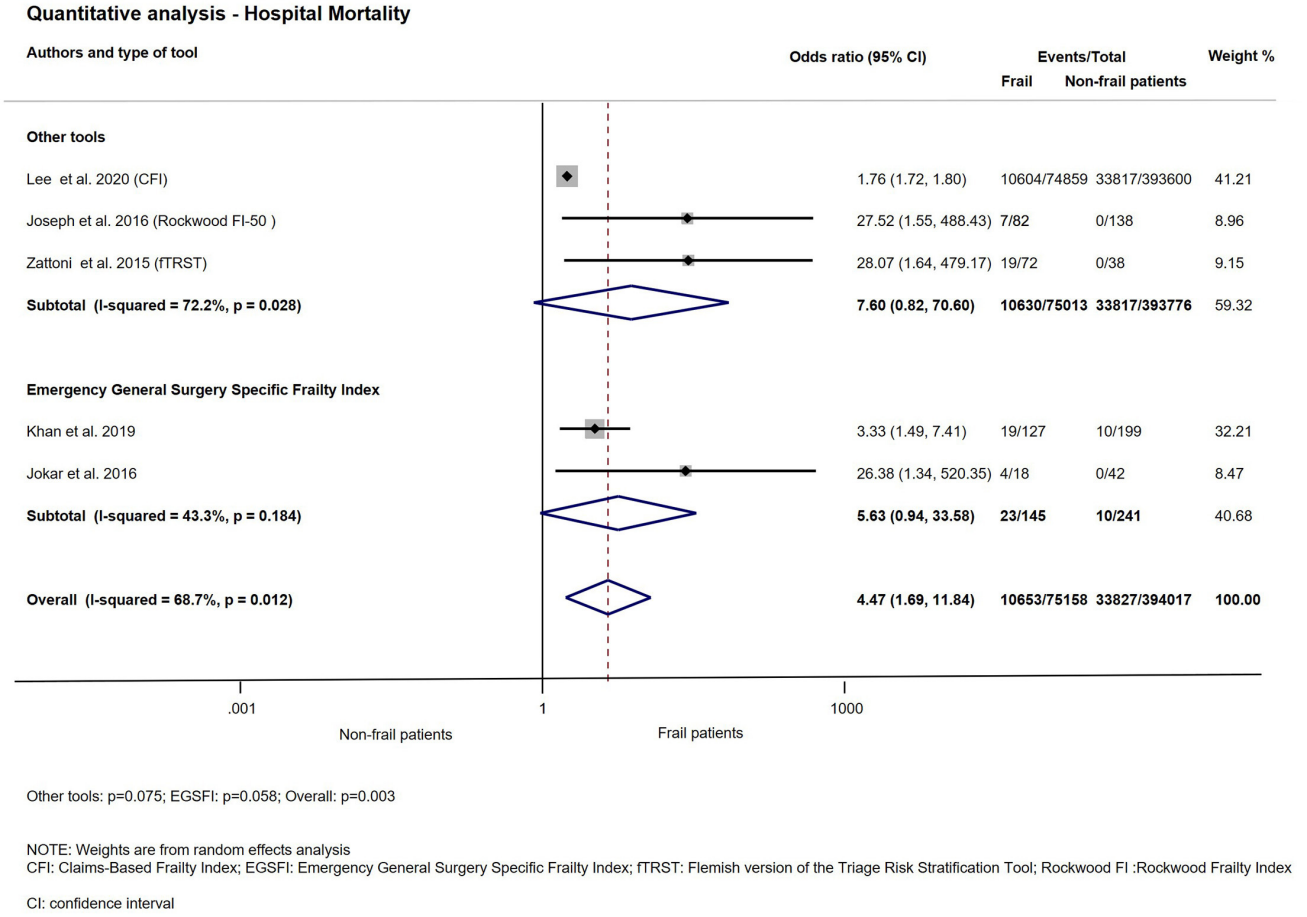
Quantitative analysis with subgroup analysis—Hospital mortality. For patients living with frailty, the overall OR of hospital mortality was 4.47; CI: 1.69–11.84. In the EGSFI subgroup frail patients have higher odds of hospital mortality (OR: 5.63; CI: 0.94–33.58). In the Other tools subgroup, the OR of hospital mortality was 7.60; CI: 0.82–70.60. Note that the overall heterogeneity and the heterogeneity for any other tools was substantial, but for EGSFI was moderate.

The association between frailty and in-hospital mortality was examined in 5 studies (24, 25, 29, 30, 37). The meta-analysis of these studies demonstrated that patients living with frailty had significantly increased chance of dying while in hospital (OR: 4.47; CI: 1.69–11.84; *p* = 0.003). The subgroup analysis of two studies which used the same assessment score, Emergency General Surgery Specific Frailty Index (EGSFI) indicated the chance of mortality while in hospital was >5-fold that of non-frail patients, but did not reach statistical significance (OR: 5.63; CI: 0.94–33.58; *p* = 0.058) (24, 29). The analysis is shown on Figure 5.

### 90-Day Mortality

**Figure 6:**
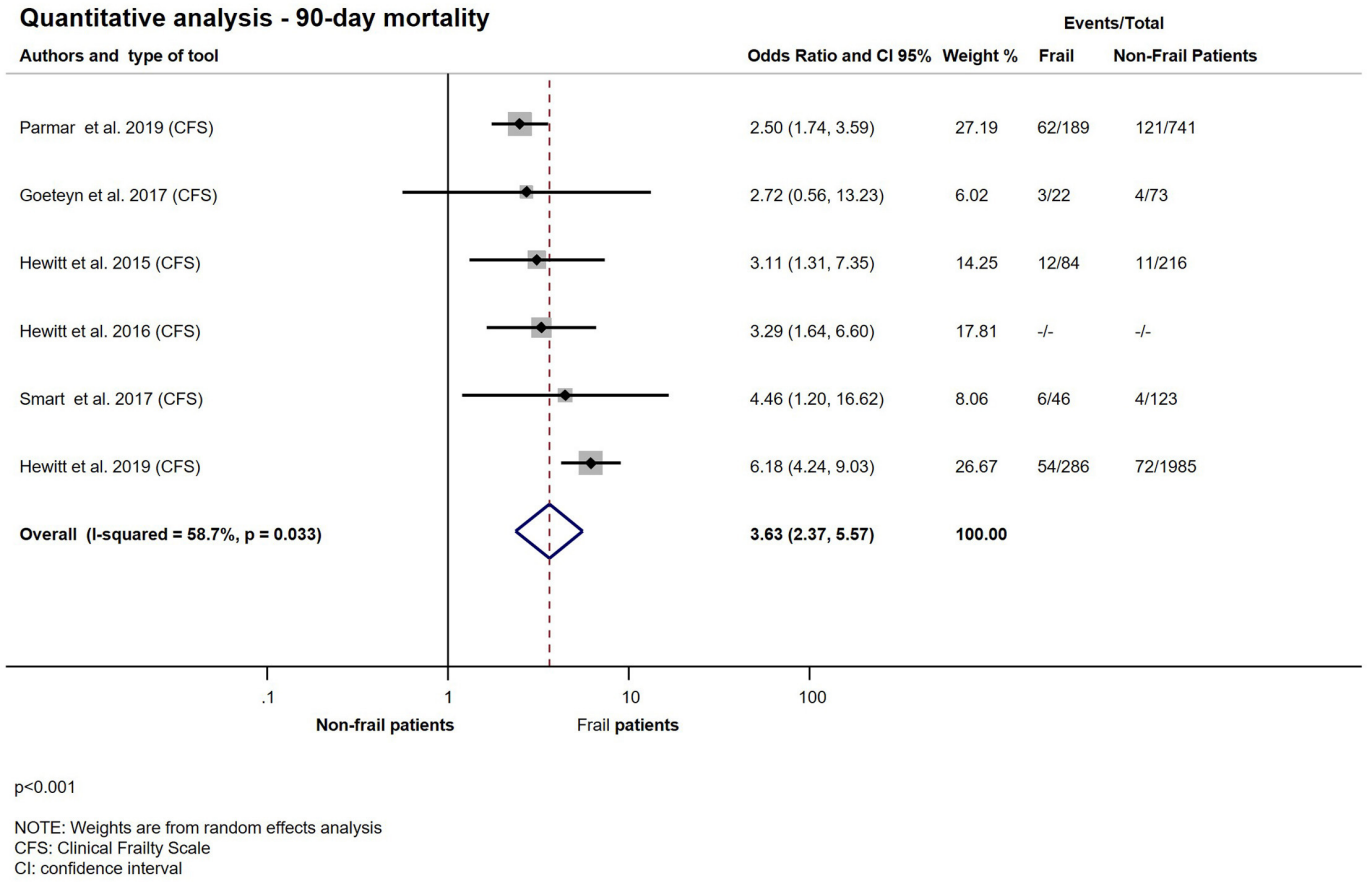
Quantitative analysis − 90-day mortality for emergency surgical patients living with frailty compared to non-frail patients. Frail patients (CFS > 4) have higher odds of 90-day mortality (OR: 3.63; CI: 2.37–5.57). Crude OR (23) was pooled with the ORs calculated from raw data. Note that heterogeneity was moderate.

The result of 90-day mortality is presented in Figure 6. All six studies used CFS for frailty assessment and reported on the 90-day mortality (10, 12, 21–23, 35). Patients living with frailty, presenting for emergency general surgery, have a 3-fold likelihood of dying within 90 days after hospital admission (OR: 3.63; CI: 2.37–5.57; *p* < 0.001).

### 12-Month Mortality

Only three studies assessed longer term (12-month) mortality among frail patients presenting for emergency surgery. All used different frailty assessment methods therefore we decided against performing quantitative analysis. Vilches-Moraga et al. reported a significantly higher 12-month mortality in association with frailty after emergency laparotomy. Mortality of patients with CFS scores of 5–9 was 59.5% compared to 28.9% with CFS 1–4 (*p* = 0.002) (34). McIsaac et al. used the Johns Hopkins Adjusted Clinical Groups frailty-defining diagnoses indicator to assess frailty. They found that frailty was associated with an increased crude hazard ratio of 1 year mortality (HR: 1.82; CI: 1.77–1.88; *p* < 0.0001) (32). Lee et al. found in a retrospective cohort that patients with moderate to severe frailty had the highest crude mortality rates, followed by those with mild frailty and pre-frailty, compared with non-frail patients (30).

### Length of Hospital Stay

**Figure 7:**
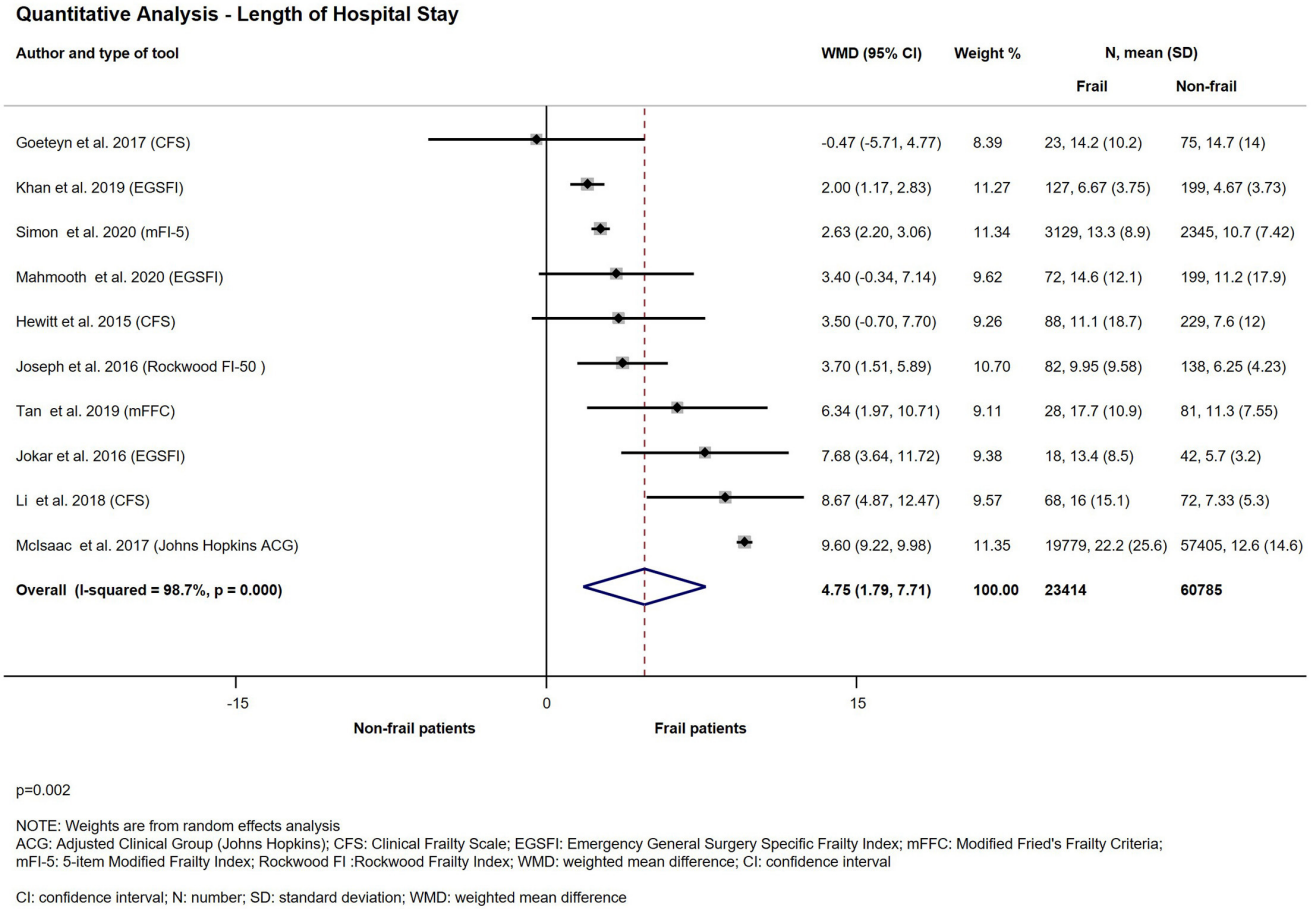
Quantitative analysis—Length of hospital stay for emergency surgical patients living with frailty compared to non-frail patients. Frail patients' average length of hospital stay was significantly higher than non-frail patients' (WMD: 4.75; CI: 1.79–7.71). Please note the considerable heterogeneity.

**Figure 8:**
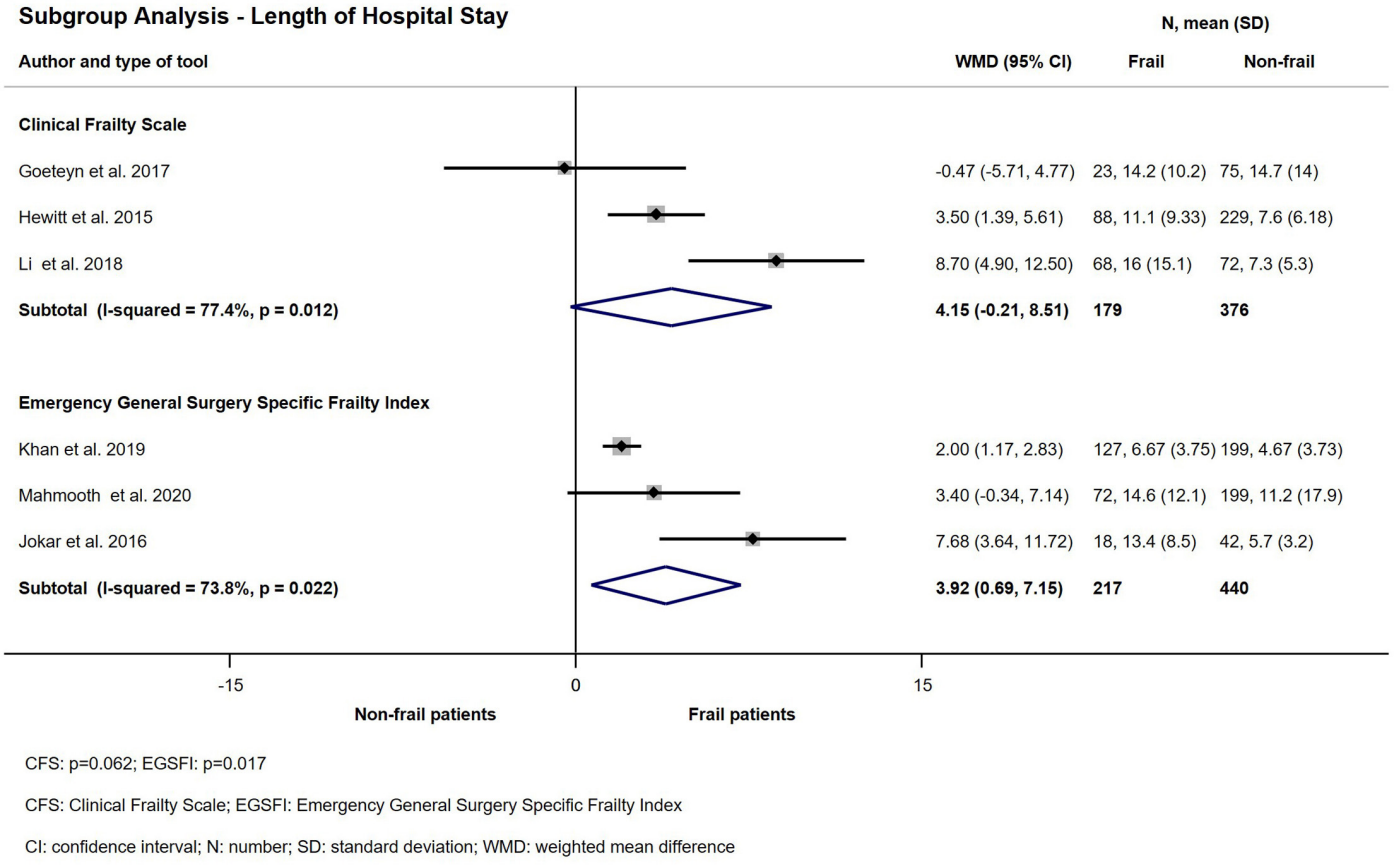
Subgroup analysis—Length of hospital stay for emergency surgical patients living with frailty compared to non-frail patients. Frail patients' average length of hospital stay was higher than non-frail patients' (CFS: WMD: 4.15; CI: −0.21–8.51) and (EGSFI: WMD: 3.92; CI: 0.69–7.15). Please note the considerable heterogeneity.

Ten eligible studies investigated the relationship between frailty and length of hospital stay (11, 21, 22, 24, 25, 29, 31–33, 36). The quantitative synthesis showed a significant difference between the length of hospitalization of non-frail patients and patients living with frailty after acute surgical admission (WMD: 4.75 days; CI: 1.79–7.71; *p* = 0.002) (Figure 7). Subgroup analysis was performed for 3 studies using CFS and another 3 using Emergency General Surgical Frailty Index as a measure of frailty (21, 22, 24, 29, 31, 33). Results of the synthesis are presented on Figure 8. In the CFS group a small prospective observational study by Goeteyn et al. reported a mean length of stay of 14.6 days which was distributed evenly in both frail and non-frail groups (*p* = 0.597) (21). On the other hand, Li et al. observed a median LOS 7 days (4–11) in the non-frail group compared to 13 days (7.5–27.5) in the frail (*p* < 0.001) (31). The pooled weighted difference in in the CFS subgroup was 4.15 days (CI: −0.21–8.51; *p* = 0.062). In the EGSFI subgroup a slightly smaller difference was detected between frail and non-frail patients in the hospital LOS (WMD: 3.92 days; CI: 0.69–7.15; *p* = 0.017).

### 30-Day Readmission

**Figure 9:**
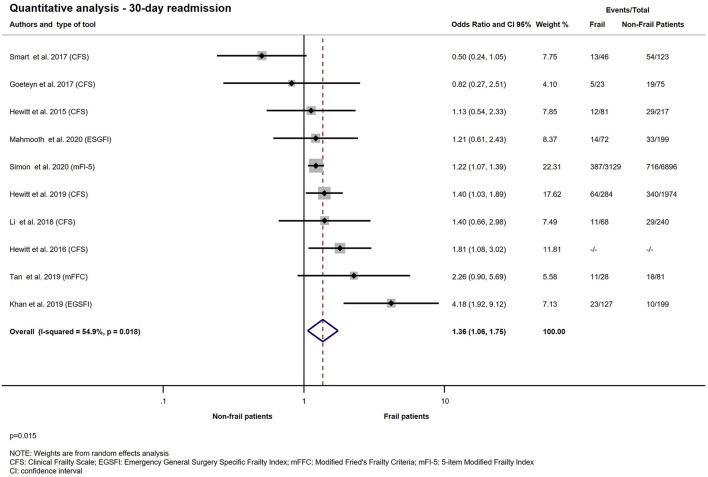
Quantitative analysis − 30-day readmission for emergency surgical patients living with frailty compared to non-frail patients. For patients living with frailty, the overall OR of 30-day readmission was 1.36 (1.06–1.75) From the study of Hewitt et al. (23) a crude OR was pooled with the ORs calculated from raw data. Note moderate heterogeneity.

**Figure 10:**
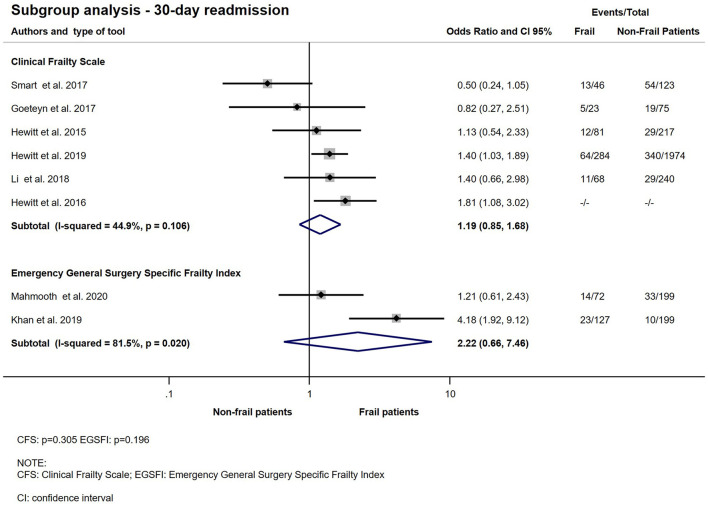
Subgroup analysis − 30-day readmission for emergency surgical patients living with frailty compared to non-frail patients. Frail patients do not have significantly higher odds of 30-day readmission (CFS: OR: 1.19; CI: 0.85–1.68) and (EGSFI: OR: 2.22; CI: 0.66–7.46). From the study of Hewitt et al. (23) a crude OR was pooled with the ORs calculated from raw data. Note that heterogeneity was moderate in the CFS group but considerable in the EGSFI group.

Ten studies with 14.273 patients reported on readmission within 30 days (11, 12, 21–23, 29, 31, 33, 35, 36). Living with frailty increased the chance of hospital readmission within 30 days after discharge following an emergency surgical hospitalization (OR: 1.36; CI: 1.06–1.75; *p* = 0.015). Quantitative analysis is presented on Figure 9. There was no significant relationship between frailty determined by CFS or EGSFI and hospital readmission rate in the emergency surgical population (CFS: OR: 1.19; CI: 0.85–1.68; *p* = 0.305 and EGSFI: OR: 2.22; CI: 0.66–7.46; *p* = 0.196). The result of the subgroup analysis of 30-day readmission is presented on Figure 10.

## Discussion

In our systematic review and meta-analysis, we examined the effects of frailty on important outcomes such as short-term mortality, readmission, and length of hospital stay in the emergency surgical population. After the selection process we included 21 studies with almost 562,000 patients regardless of their age, although most of them were older than 65 years at the time of their hospital admission. While 18 different frailty assessment methods were used in the reviewed studies, not surprisingly, the most widely used was the 7-point version of the Clinical Frailty Scale.

We have shown that patients living with frailty have an increased chance of hospital-, 30- and 90-day mortality after an emergency surgical hospital admission compared to non-frail patients. While we decided against doing quantitative analysis on 12- month mortality due to low number of studies with different frailty assessment tools, the reviewed literature strongly suggests that patients living with frailty are more likely to die during the first post-operative year.

Two previous meta-analyses published in the past 5 years investigated frailty in emergency surgical patient group (38, 39). Although our results are in line with their conclusion, there are some significant differences in the methodology. Fehlmann et al. demonstrated a significant association between frailty and unfavorable outcomes after emergency general surgery. In their review frailty was measured by the Clinical Frailty Scale or the Modified Frailty Index (mFI). They included 3 studies, all used CFS in the quantitative analysis. They only included adults ≥65 years of age, who underwent surgical procedures. There is evidence that frailty affects not just the older generation but younger as well (12, 35). In a large cohort of young trauma patients (age < 60) Rege et al. reported that frailty assessed by mFI-5 was an excellent predictor of thirty-day mortality (OR: 11.02; CI: 6.26–19.39; *p* < 0.001), better than age (OR: 1.066; CI: 1.059–1.072) and ASA class (OR: 2.75; CI: 2.50–3.01 *p* < 0.001) (40). Smart et al. found in a small, prospective cohort of emergency general surgical patients that frailty was present in the younger group and resulted in not just longer hospital stay but 5-fold greater risk of mortality at 30 and 90 days (OR: 5.67; CI: 0.33–96.89) (35). Therefore, the exclusion of the younger patient cohort from their review may limit the generalizability of its findings. In the other meta-analysis, Ward et al. demonstrated that frailty is associated with adverse outcomes (38). They could only include 7 studies to their systematic review and 4 studies with 55.193 patients to the meta-analysis. They pooled together studies with different frailty assessment methods as a consequence considerable heterogeneity was observed. The combined risk of mortality within 30 days in frail patients was higher than non-frail patients (Relative Risk: 3.04; CI: 2.67–3.46; *p* < 0.01; Cochrane Q < 0.01; *I*^2^ = 76%). On analyzing our results, although we were able to include 14 studies with 483.722 participants, we observed the same degree of heterogeneity when all different methods were pooled together. Conversely heterogeneity might not be important in the CFS subgroup (*I*^2^=8.7%; *p* = 0.363). Fehlmann et al. reported a significant association between frailty and length of hospital stay (adjusted ORs were 1.21, 1.26, 1.48, 1.44, and 1.62 for CFS 2, 3, 4, 5, and 6–7, respectively) (39). When we analyzed all the available data, we also found significant relationship between frailty and length of hospital stay. In our subgroup analysis although there is a tendency of increased LOS in the emergency surgical patient population with frailty when either the CFS or the EGSFI was used, however, due to the limited number of patients and studies firm conclusions cannot be drawn.

Several studies investigated the relationship between frailty and the need for readmission in a wide variety of patient groups. On the one hand Vidan et al. found that frail elderly patients (≥70 years old) hospitalized for heart failure had a similar rate of 30-day readmission compared to non-frail patients (17.4 vs. 15.1%; *p* = 0.74) (41). On the other hand, a study enrolling patients with inflammatory bowel disease reported a 21% higher risk of all-cause readmission (adjusted hazard ratio: 1.21; CI: 1.17–1.25) in the frail patient group (42). Furthermore in a systematic review of elderly patients undergoing elective surgery for colorectal cancer Fagard et al. reported increased need for readmission in the frail group (43). In our quantitative analysis of 10 studies although we have found significant association between readmission and frailty in the emergency surgical patient group, very high heterogeneity was observed (*I*^2^ = 98.7 %; *p* < 0.001).

Although frailty is present in the younger age group, the majority of patients who are living with frailty are older than 65 years. This population group represents higher proportion of most western societies than earlier during the twentieth century and is predicted to increase further. It is due to falling fertility rate and increasing life expectancy. Based on this we do not need a crystal ball to forecast an increase of frail emergency surgical admissions. Additionally, the COVID 19 pandemic caused huge number of cancellations of elective surgical work and it is highly likely that some of the postponed elective surgical patients will present as emergencies and anesthetist and surgeons will need to deal with more frail complex patients than before.

Based on our findings assessing frailty and incorporating it into risk prediction tools instead of age may improve prognostication in this patient group. More importantly creating pathways and protocols based on frailty for emergency surgical patients can potentially improve outcome and mitigate some of the increased chance of dying and other unfavorable consequences such as need for readmission and prolonged hospital stay.

### Strength and Limitations

We believe our work is the most comprehensive review and analysis of the literature to date, including patients of younger age and the highest variety of validated frailty scores. Furthermore, we were able to pool together studies using the same frailty score and present subgroup analysis. Unfortunately, some assessment tools were only used by single research group, therefore, despite our extensive search we were only able to perform subgroup analysis on studies which used Clinical Frailty Scale, Triage Risk Stratification Tool Emergency General Surgery Specific Frailty Index and: Geriatric screening tool (G8). Due to the relatively inclusive stance of our selection criteria (patients aged over 18 years old and admitted to hospital with a general surgical complaint, including those undergoing surgery and those managed conservatively) high heterogeneity was observed in many of the analyses. Presence of confounding bias in most of the analyzed studies may also limits the strength of our findings.

## Conclusion

Our meta-analysis has demonstrated that frailty is a good marker for ultimately poor outcome and may also be associated with prolonged hospital stay and need for readmission but further research is needed to investigate how it might identify patients who could somehow be optimized if it was known preoperatively.

## Implication for Practice and Research

Frailty potentially a very important factor but further research needs to be done to assess the benefits and costs of frailty screening for emergency surgical patients. Furthermore, comprehensive assessment of the different frailty scales is needed, and frailty-based pre-emptive interventions should be tested in clinical trials.

## Data Availability Statement

The original contributions presented in the study are included in the article/Supplementary Material, further inquiries can be directed to the corresponding author/s.

## Author Contributions

TL performed the systematic search and selection, data extraction, risk of bias assessment, prepared the figures and tables, and wrote the manuscript. DN performed the statistical analysis and wrote the statistical part of the methods section of the manuscript. PH, MR, and MVa provided expert opinion during the writing of the manuscript. KO provided methodological counsel in all phases from preliminary searches to writing of the manuscript. MVi helped to perform the systematic search and selection, data extraction, and risk of bias assessment. SK provided methodological counsel in the systematic search, risk of bias assessment, and helped in the writing of the manuscript. AV was our senior biostatistician and helped to perform the statistical analysis. ZM coordinated the work and provided expert opinion. All authors contributed to the article and approved the submitted version.

## Funding

This work was supported by the Hungarian National Research, Development and Innovation Office (Grant No: K 138816).

## Conflict of Interest

The authors declare that the research was conducted in the absence of any commercial or financial relationships that could be construed as a potential conflict of interest.

## Publisher's Note

All claims expressed in this article are solely those of the authors and do not necessarily represent those of their affiliated organizations, or those of the publisher, the editors and the reviewers. Any product that may be evaluated in this article, or claim that may be made by its manufacturer, is not guaranteed or endorsed by the publisher.
