# Supplementary material for: Frailty and Emergency Surgery: Results of a Systematic Review and Meta-Analysis
Source: Front Med (Lausanne). 2022 Mar 31;9:811524. doi: 10.3389/fmed.2022.811524 (PMC9008569; doi:10.3389/fmed.2022.811524)
Supplement: Supplementary file 1 [file Data_Sheet_1.docx]

**Supplementary materials**

# **PICO/PECO**

P- Adults admitted to hospital with acute (emergency) surgical problem

E- Frailty as indicated by scoring

C- Non-frail patients

O- 30-days mortality, 90-days mortality, hospital mortality, 12-month mortality, length of hospital stay, and 30 days hospital readmission

# **Risk of bias assessment protocol**

Following the recommendations of the Cochrane Collaboration, the Quality in Prognosis Studies (QUIPS) tool was used by TL and MV independently (1). Any disagreement was resolved based on consensus. In the study participation domain gender, age, ethnicity and comorbidities were taken into account. Study attrition was not judged for retrospective studies. In the prognostic factor measurement domain, the specification of the frailty assessor, information about their training and missing data on frailty were taken into account. Less than 10% missing data was considered low risk, 10-20% some concerns and more than 20% resulted in high risk for the whole domain. Outcome measurement and statistical analysis domains carried low risk in most cases because mortality is a hard outcome and we mostly used raw data. In the study confounding domain, studies reporting baseline information for the frailty groups separately were judged low risk if no clinically significant differences were seen, some concerns if some differences were seen and high risk if no data was reported. The overall RoB was calculated using the suggestions of Grooten et al. (2).

# **Figure S1**


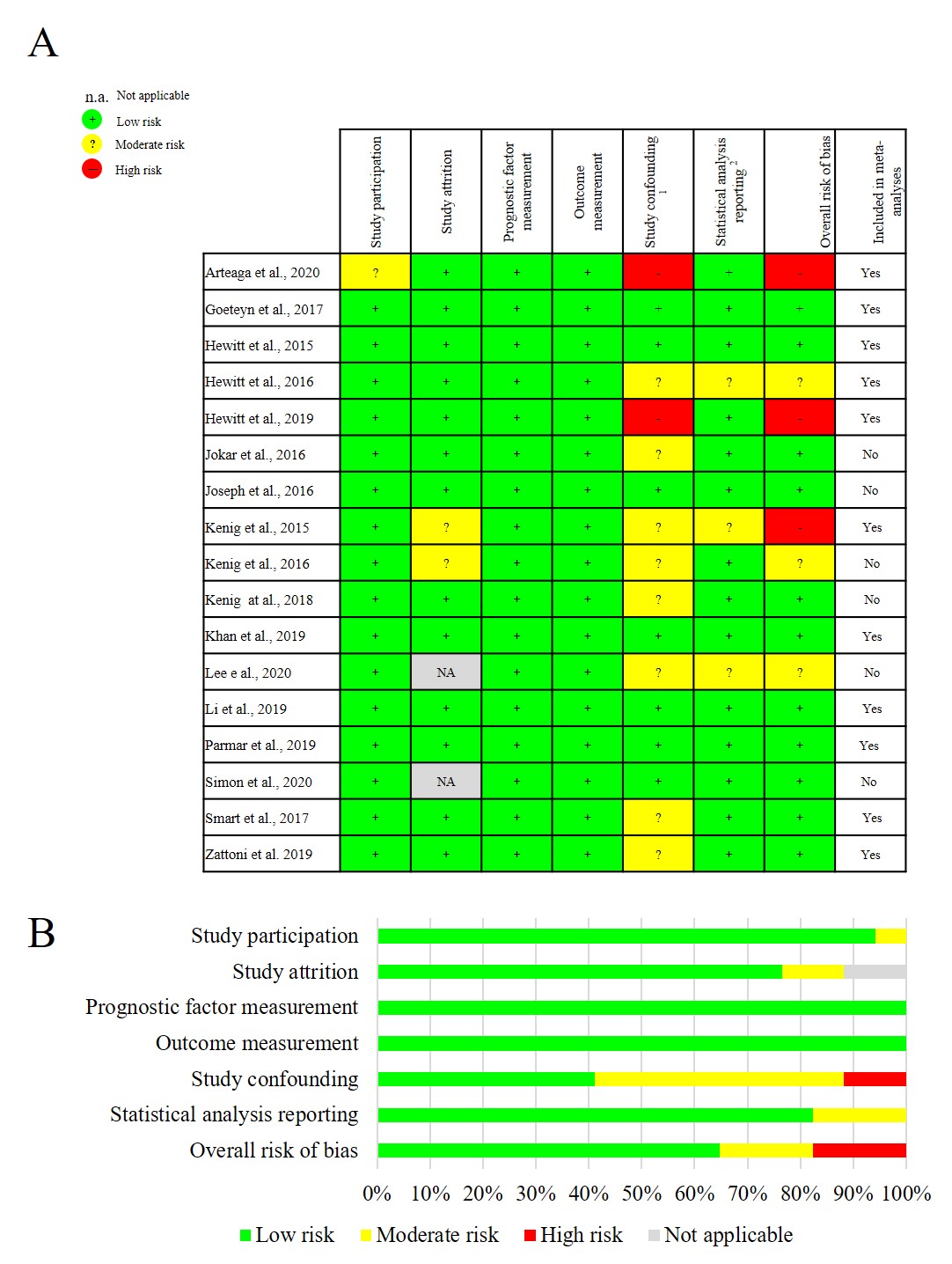


**Figure S1 Risk of bias assessment on study level [A] and across studies [B] for studies reporting 30-days, 90-days, and in-hospital mortality in frail versus not frail patients**
For details please see the protocol for risk of bias assessment above.

# **Figure S2**

#
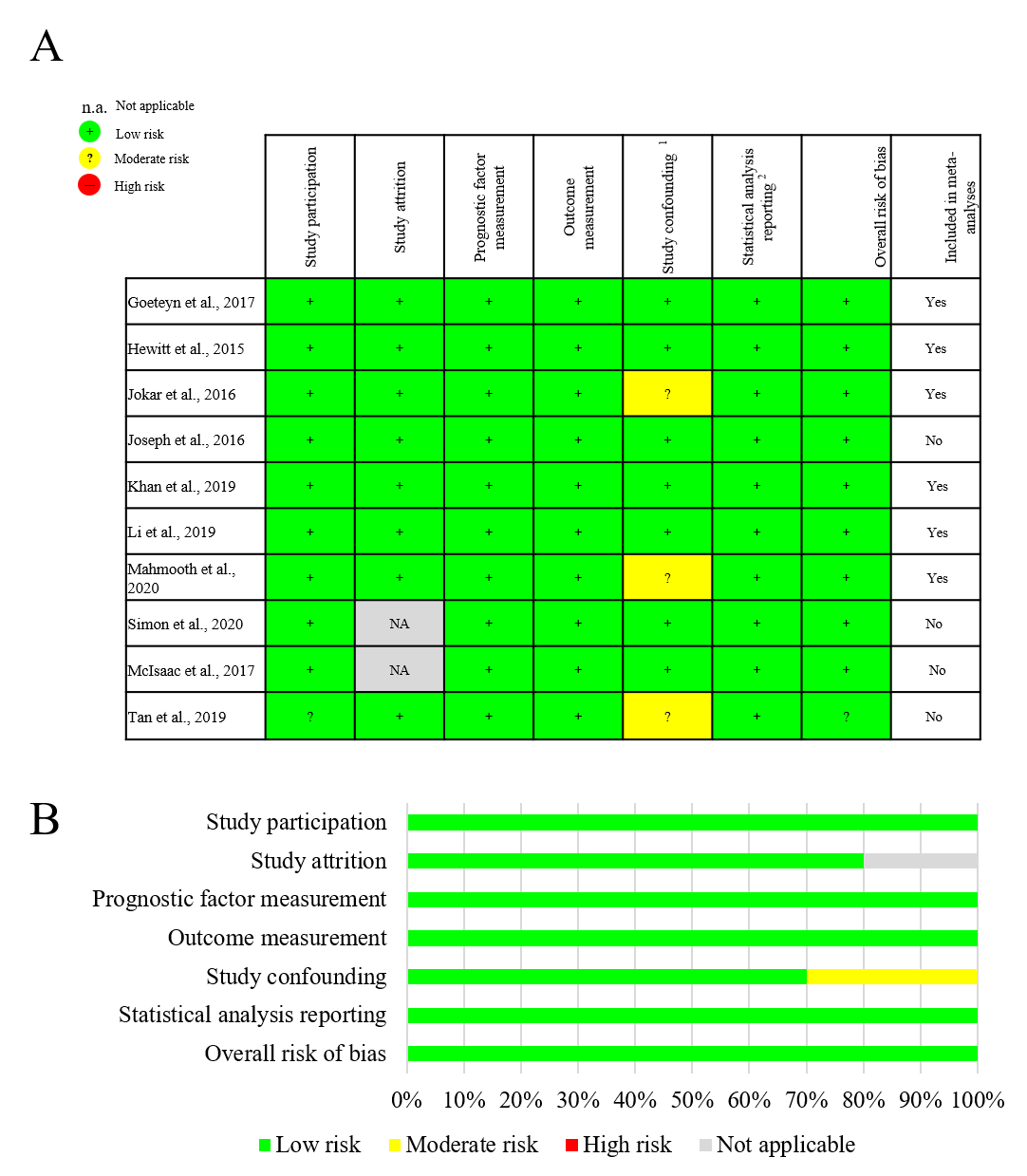


**Figure S2 Risk of bias assessment on study level [A] and across studies [B] for studies reporting 30-day readmission in frail versus not frail patients**
For details please see the protocol for risk of bias assessment above.

# **Figure S3**


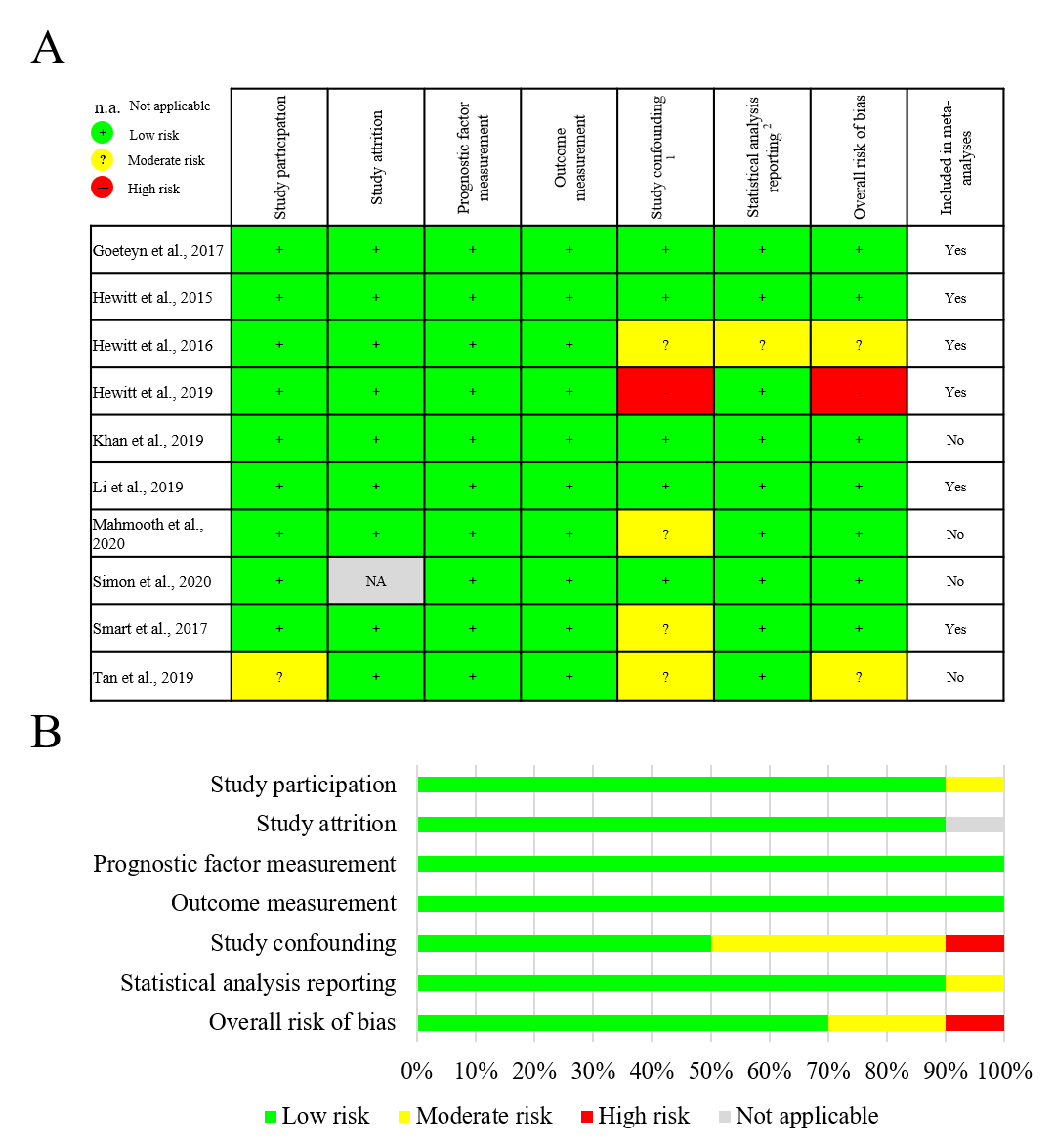


**Figure S3 Risk of bias assessment on study level [A] and across studies [B] for studies reporting length of hospital stay in frail versus not frail patients**

For details please see the protocol for risk of bias assessment above.

**References**

1. Hayden JA, Côté P, Bombardier C. Evaluation of the Quality of Prognosis Studies in Systematic Reviews. *Annals of Internal Medicine* (2006) 144(6):427-37. doi: 10.7326/0003-4819-144-6-200603210-00010.

2. Grooten WJA, Tseli E, Äng BO, Boersma K, Stålnacke B-M, Gerdle B, et al. Elaborating on the assessment of the risk of bias in prognostic studies in pain rehabilitation using QUIPS—aspects of interrater agreement. *Diagnostic and Prognostic Research* (2019) 3(1):5. doi: 10.1186/s41512-019-0050-0.
